# Zinc portioning and allocation patterns among various tissues confers variations in Zn use efficiency and bioavailability in lentil genotypes

**DOI:** 10.3389/fpls.2023.1325370

**Published:** 2024-01-29

**Authors:** Naser Rasheed, Muhammad Aamer Maqsood, Tariq Aziz, Muhammad Imran Ashraf, Ifra Saleem, Shabana Ehsan, Allah Nawaz, Hafiz Muhammad Bilal, Minggang Xu

**Affiliations:** ^1^Department of Soil Science, University of Agriculture, Faisalabad-Sub Campus Depalpur, Okara, Pakistan; ^2^Institute of Soil and Environmental Sciences, University of Agriculture, Faisalabad, Faisalabad, Pakistan; ^3^Soil Chemistry Section, Ayub Agricultural Research Institute, Faisalabad, Pakistan; ^4^Soil Bacteriology Section, Ayub Agricultural Research Institute, Faisalabad, Pakistan; ^5^Water Management Research Farm (WMRF), Renala Khurd, Agriculture Department, On Farm Water Management, Punjab, Lahore, Pakistan; ^6^Shanxi Key Laboratory of Soil Environment and Nutrient Resources, Shanxi Institute of Ecological and Environmental Technology, Shanxi Agricultural University, Taiyuan, China

**Keywords:** lentil, Zn accumulation, cultivars, partitioning, malnutrition, bioavailability

## Abstract

Zinc (Zn) is essential for plants and animals as it plays significant roles in several physiological and biological processes. Its deficiency in soil results in low Zn content food and is one of the major reasons for Zn malnutrition in humans. Biofortification of crops with zinc (Zn) is a viable approach to combat malnutrition, especially in developing countries. A hydroponic study was executed to study response and Zn partitioning in various lentil genotypes. Eight preselected lentil genotypes (Line-11504, Mansehra-89, Masoor-2006, Masoor-85, Line-10502, Markaz-09, Masoor-2004, and Shiraz-96) were grown in solution culture with two Zn levels (control and adequate Zn). Plants were sown in polythene lined iron trays with a two inch layer of prewashed riverbed sand. After 10 days of germination, seedlings were transplanted to a 25L capacity container with nutrient solution for 15 days, and afterward, these plants were divided into two groups, receiving either 2.0 mM Zn or no Zn levels. Three plants of each genotype were harvested at the vegetative growth stage (60 DAT) and the remaining three at physiological maturity (117 DAT). Plants were partitioned into roots, shoots, and grains at harvest. Significant variations in root and shoot dry matter production, grain output, partitioning of Zn in plant parts (root, shoot, and grain), grain phytate reduction, and Zn bioavailability were observed among genotypes. Lentil root accumulated more Zn (54 mg kg-1) with respect to shoot Zn (51 mg kg-1) under Zn supply. The Zn efficient genotypes (Line-11504 and Mansehra-89) produced more root and shoot dry weights at both harvests. There was a positive correlation between the relative growth rate of root and grain phytate concentration (r = 0.55) and [phytate]:[Zn] ratio (r = 0.67). Zn-efficient genotype Mansehra-89 had a maximum root shoot ratio (0.57) and higher grain Zn (60 mg kg-1) with a respectively reduced grain phytate (17 µg g-1) and thus, had more Zn bioavailability (3.01 mg d-1). The genotypic ability for Zn uptake and accumulation within different plant tissues may be incorporated into future crop breeding to improve the nutrition of undernourished consumers.

## Introduction

Zinc (Zn) is an essential nutrient for living organisms because of its role in the growth and development of the immune system, its activation of enzymes, proteins, and DNA synthesis, which are important for the development of neurobehavior (Grusak, 2009; Tuso et al., 2013; Singh et al., 2016). The World Health Organization (WHO) and Food and Agriculture Organization (FAO) recommend 4.9 mg and 7 mg of Zn for males and females aged 19-50 years of age, respectively (based on 10% bioavailability) (Grusak, 2009). Zinc malnutrition in humans is widespread, especially in developing countries like South Asia (29.6%) and sub-Saharan Africa (25.6%) (Singh et al., 2016). This deficiency is related to the intake of low Zn content food (i.e. cereal based diets) and higher contents of anti-nutrients like phytates, as these compounds reduce Zn bioavailability. Increasing Zn content and bioavailability in food through agronomic or genetic measures, which may overcome widespread malnutrition in these countries (White and Broadley, 2009; Bouis and Welch, 2010; Khush et al., 2012; Hussain et al., 2013; Zaman et al., 2018). Food supplementation, fortification, and biofortification are the three most feasible approaches to address widespread Zn deficiency in humans. The first two approaches require healthcare systems, infrastructure, and uninterrupted funding: all of which are often limited in developing countries. Therefore, the biofortification (sustainable approach for improving the dietary quality and alleviating zinc deficiency globally) of Zn in grains, an efficient strategy to combat hidden hunger (Cakmak, 2008; Cakmak et al., 2010; Bouis et al., 2011; Bouis and Saltzman, 2017; Dwivedi et al., 2023).

The Zn content in food grains can be increased by applying Zn to crops, which also improves grain yield (Kaya et al., 2009; Nasri et al., 2011; Cakmak and Kutman, 2018; Rehman et al., 2018; Rasheed et al., 2020). Crop genotypes also differ significantly in Zn uptake from soil and also in the translocation of Zn from roots, leaves, and stems to edible portions i.e. grains (Cakmak et al., 2004; Grusak and Cakmak, 2005; Hussain et al., 2013; Maqsood et al., 2015), which mainly depends on the plant growth stage(s) and xylem loading, etc. Variations in the concentration of Zn and differences in the extent of the response towards applied Zn have been observed and reported in various crops like chickpeas (Diapari et al., 2014), peas, common beans, and lentils (Ray et al., 2014), wheat (Maqsood et al., 2015), maize (Rasheed et al., 2019), etc. Moreover, an increase in the uptake and translocation of Zn to shoot has also been observed in solution culture (Rasheed et al., 2019), which in turn increased the grain output and Zn concentration (Hambidge, 2000; Hoffland et al., 2006; Pandey et al., 2006; Pandey et al., 2009), but their mechanisms (partitioning and allocation pattern) are not understood well. Zinc accumulation in grains may not only be related to root uptake but may be dependent on the internal remobilization and redistribution within the different plant parts, which mostly occurs through transport in the xylem, transfer from the xylem to the phloem, and re-translocation in the phloem (Jiang et al., 2007). Therefore, there is a dire need to investigate the partitioning and allocation pattern of uptake Zn, so that more Zn efficient/fortified genotypes may be developed to feed undernourished people of the world, especially in developing countries.

Lentil (*Lens culinaris* Medik) is one of the most popular types of pulses due to its relatively quick cooking time, and low cost, with high-quality protein, vitamins, dietary fiber, and minerals, e.g., iron, zinc, selenium, etc. (Reddy, 2001; Thavarajah et al., 2011; Mao et al., 2014; Velu et al., 2014; Singh, 2017). The nutritional profile of lentil seeds is recognized as a good food source for human nutrition across the world. Thus, lentils can be supplemented with cereals to improve food quality and affordability in developing and underdeveloped countries. Moreover, significant genetic variations in lentil germplasm have been observed for Zn use efficiency and recently we categorized local genotypes as efficient and responsive vs inefficient and non-responsive (Brennan et al., 2001; Pathak et al., 2012; Rasheed et al., 2020). The present experiment was planned to identify the responsible mechanisms of these genetic variations particularly the Zn partitioning (translocation, and distribution) into different tissues, especially into grains of pre-identified efficient and inefficient Zn genotypes. The differences in the various parameters of these genotypes could help us identify the major mechanisms involved enabling them to be incorporated in future breeding programs.

## Materials and methods

### Plant material and growth condition

We obtained the seeds from the National Agriculture Research Council (NARC), Islamabad, Pakistan, and the Ayub Agriculture Research Institute (AARI), Faisalabad, Pakistan. The seed genotypes were selected from previous experiments (Rasheed et al., 2020), including: a) Zn efficient, Line-11504 and Mansehra-89; b) Zn inefficient, Masoor-2006 and Masoor-85; c) Zn responsive, Line-10502 and Markaz-09; d) Zn non-responsive, Masoor-2004 and Shiraz-96 The seeds were sown in polythene lined iron trays with a two-inch layer of prewashed riverbed sand at the Institute of Soil & Environment Sciences rain-protected greenhouse of the University of Agriculture, Faisalabad (location 73.072058° North and 31.433518° East). After 10 days of germination seedlings were transplanted to a 25L capacity container with nutrient solution (Johnson et al., 1957 modified by Aziz et al., 2014). The composition of the full-strength nutrient solution (pH 6.5) was 5 *m*M nitrogen (N), 0.2 *m*M phosphorus (P), 3.5 *m*M potassium (K), 1.5 *m*M calcium (Ca), 0.5 *m*M magnesium (Mg), 2.05 *m*M sulfur (S), 50 *μ*M chloride (Cl), 25 *μ*M boron (B), 2 *μ*M manganese (Mn), 0.5 *μ*M copper (Cu), 0.5 *μ*M molybdenum (Mo), 50 *μ*M iron (Fe). Iron was used as Fe-ethylene diamine tetra acetic acid (EDTA). Plants were grown for 15 days with adequate Zn i.e. 2.00 *μ*M Zn. The plants were then divided into two groups, the first received adequate Zn (2.00 *μ*M) and the other received no Zn. The nutrient solution was replaced with a fresh nutrient solution weekly to ensure a continuous supply of nutrients. The pH of the solution was monitored (pH meter, Make Hanna, Model HI 98128) and maintained daily at 6.5 ± 0.2 with 1*N* HCl or 1*N* NaOH. Three plants of each genotype were harvested at the vegetative growth stage (60 DAT) and the remaining three plants were harvested at physiological maturity (117 DAT). Plants were separated into roots, shoots, and grains for various physiological growth parameters as well as Zn and phytic acid concentration analysis.

### Plant growth parameters

The collected samples of roots and shoots were air dried and then oven dried at 65°C for 48 h in a forced air-driven oven and oven-dried weights were measured for each harvest. The root-to-shoot ratio was calculated on a dry weight basis. Root and shoot relative growth rates (RGR) were calculated as in Equation 1.


(1) $$RGR=\frac{In\;W2-In\;W1}{\left(t2-t1\right)}$$

where W1 and W2 are plant dry weights at times t_1_ and t_2_ (60 and 117 DAT) (Hoffmann and Poorter, 2002).

### Zn and phytate analysis in lentil

The oven dried samples were ground to fine powder in a stainless-steel grinder. Finely ground samples (0.2 g each) were digested in di-acid mixture using HNO_3_:HClO_4_ (2:1) and the final volume was up to 25 ml (Jones and Case, 1990). The concentration of Zn in digested samples was analyzed by using an atomic absorption spectrophotometer. For the determination of the phytate, extraction of samples (60 mg) was made with 10 mL solution (0.2 N HCl) for 2 hours by shaking at room temperature. The indirect method was used for phytate determination in which 2,2′-bi-pyridine and un-reacted Fe (III) (Haug and Lantzsch, 1983) were used to develop a pink color and their absorption was measured at 519 nm with a spectrophotometer. Zinc bioavailability in grains of lentils was qualitatively determined by phytate to Zn molar ratio employing by trivariate model for absorption of Zn (Hambidge et al., 2010; Hussain et al., 2013; Rasheed et al., 2020) (Equation 2). The use of the mathematical Zn absorption model explains the quantitative measurements of Zn bioavailability by utilizing the absorption of Zn into the body (Miller et al., 2007).


(2) $$TAZ=0.5\cdot \left({\text{A}}_{\mathrm{MAX}}+\mathrm{TDZ}+{\text{K}}_{\text{R}}\cdot (1+\frac{\mathrm{TDP}}{{\text{K}}_{\text{P}}})-\sqrt{{\left({\text{A}}_{\mathrm{MAX}}+\mathrm{TDZ}+{\text{K}}_{\text{R}}\cdot (1+\frac{\mathrm{TDP}}{{\text{K}}_{\text{P}}})\right)}^{2}-4\cdot {\text{A}}_{\mathrm{MAX}}+\mathrm{TDZ}}\right)$$

The parameters defined as, AMAX “the maximum absorption”, KR “equilibrium dissociation constant of Zn-receptor binding reaction” and KP “equilibrium dissociation constant of Zn-phytate binding reaction” (Hambidge et al., 2010), which relates to the homeostasis of Zn in human’s intestine and the AMAX, KP, and KR have constant values of, 0.091, 0.033 and 0.680, respectively. This model measures the TAZ (total daily absorbed Zn in mg Zn d^−1^) based on TDZ (total daily dietary Zn) in mmol Zn d^−1^ and TDP (total daily dietary phytate) in mmol phytate d^−1^.

### Statistical analysis

Data regarding plant growth, root and shoot dry matter production, Zn concentration, and other related parameters of Zn bioavailability were subjected to analysis of variance (ANOVA) using STATISTICS 8.1 computer-based program. Differences in genotype means were compared statistically using Tukey’s HSD test (Honestly Significant Difference) at a 5% probability level (Steel et al., 1997). Relationships between different parameters were also measured using the Pearson correlation coefficient.

## Results

### Variation in plant growth and yield

#### Biomass yield

There were significant main and interactive effects (Treatment x Genotype) on root dry weight (RDW), shoot dry weight (SDW), and root:shoot ratio (RSR) at both harvests (**Tables 1**, **2**). At 60 DATharvest Zn efficient genotype Mansehra-89 produced about 3 fold more RDW than Zn non-responsive genotype Masoor-2004 in treatment receiving no Zn application. The application of Zn improved both RDW and SDW in all genotypes (**Tables 1**, **2**), though the increase varied significantly among genotypes. A maximum increase in RDW was observed in Zn responsive genotype Line-10502, than in Zn inefficient genotype Masoor-85. A higher value of shoot dry weight (3.35 g plant^-1^) was obtained in the Zn-efficient genotype (Mansehra-89) with Zn application, whereas the minimum shoot dry weight (1.5 g plant^-1^) was observed in the Zn non-responsive genotype Masoor-2004 (**Table 1**). In the case of SDW, the Zn efficient genotype (Line-11504) showed a maximum increase (56%) under Zn supply, while the least response was observed in Masoor 85 (Zn inefficient) in 60 DAT harvest. While at maturity, the highest SDW (6.43 g plant^-1^) was produced by Mansehra-80 (Zn-efficient) with Zn application, and the lowest SDW (2.81 g plant^-1^) was observed in Shiraz-96 (Zn non-responsive genotype). There was a 2-fold increase in shoot dry matter production, which was observed in the Zn-efficient genotype (Line-11504) with Zn application.

**Table 1:** Plant growth and yield paraments of lentil genotypes as influenced by solution Zn application at different growth stages.

Genotypes	Treatments	Harvest at 60 DAT		Harvest at 117 DAT				
		Root Dry Weight	Shoot Dry Weight	Root Dry Weight	Shoot Dry Weight	No. of Pods	100 Seed Weight	Grain Yield
		g plant^-1^	g plant^-1^	g plant^-1^	g plant^-1^	plant^-1^	g	g plant^-1^
**Line-10502**	**Control**	0.58 ± 0.06	1.7 ± 0.06	1.03 ± 0.18	3.97 ± 0.23	73 ± 7.21	1.71 ± 0.12	2.11 ± 0.32
	**With Zn**	1.14 ± 0.09	2.52 ± 0.07	1.34 ± 0.19	5.24 ± 0.43	111 ± 17.04	1.85 ± 0.09	2.79 ± 0.18
**Markaz-09**	**Control**	0.86 ± 0.05	2.15 ± 0.08	0.99 ± 0.28	3.59 ± 0.47	70 ± 6.11	1.74 ± 0.11	2.07 ± 0.21
	**With Zn**	1.27 ± 0.06	3.09 ± 0.08	1.37 ± 0.23	5.2 ± 0.23	100 ± 14.18	1.84 ± 0.1	2.46 ± 0.32
**Masoor-2006**	**Control**	0.62 ± 0.04	2.23 ± 0.1	0.91 ± 0.03	3.62 ± 0.26	78 ± 11.79	1.69 ± 0.1	1.9 ± 0.21
	**With Zn**	0.98 ± 0.08	2.82 ± 0.08	1.20 ± 0.22	4.69 ± 0.21	118 ± 7.51	1.82 ± 0.12	2.86 ± 0.34
**Line-11504**	**Control**	0.97 ± 0.09	1.96 ± 0.08	1.1 ± 0.15	3.31 ± 0.48	88 ± 11.37	1.83 ± 0.13	2.91 ± 0.35
	**With Zn**	1.44 ± 0.15	3.06 ± 0.07	1.49 ± 0.09	6.07 ± 0.33	148 ± 10.82	2.04 ± 0.2	4.97 ± 0.61
**Masoor-85**	**Control**	0.74 ± 0.08	2.35 ± 0.06	1.06 ± 0.19	3.91 ± 0.38	68 ± 11.53	1.57 ± 0.14	1.85 ± 0.23
	**With Zn**	0.95 ± 0.07	2.68 ± 0.06	1.24 ± 0.17	5.15 ± 0.46	101 ± 13.05	1.64 ± 0.14	2.61 ± 0.65
**Mansehra-89**	**Control**	1.3 ± 0.12	2.28 ± 0.07	1.37 ± 0.08	4.77 ± 0.25	103 ± 22.87	1.8 ± 0.07	2.53 ± 0.31
	**With Zn**	1.53 ± 0.26	3.35 ± 0.06	1.68 ± 0.04	6.43 ± 0.52	150 ± 9.81	2.1 ± 0.18	5.23 ± 0.37
**Masoor-2004**	**Control**	0.49 ± 0.06	1.5 ± 0.05	0.92 ± 0.16	3.73 ± 0.5	81 ± 15.62	1.68 ± 0.04	2.19 ± 0.33
	**With Zn**	0.83 ± 0.06	1.98 ± 0.08	1.23 ± 0.17	4.69 ± 0.46	105 ± 23.52	1.78 ± 0.09	2.61 ± 0.62
**Shiraz-96**	**Control**	0.68 ± 0.08	1.98 ± 0.09	1.04 ± 0.09	2.81 ± 0.37	91 ± 10.02	1.65 ± 0.09	1.83 ± 0.29
	**With Zn**	1.09 ± 0.08	2.65 ± 0.06	1.26 ± 0.07	4.91 ± 0.45	110 ± 25.5	1.76 ± 0.07	2.4 ± 0.16

Values are presented as mean ± SD. DAT, days after transplantation.

**Table 2:** Mean square values for different plant parameters of lentil genotypes under solution Zn application.

Source	DF	*60 DAT Harvest*			*117 DAT Harvest*				
		*Root Dry Weight*	*Shoot Dry Weight*	*RSR*	*Root Dry Weight*	*Shoot Dry Weight*	*RSR*	*Root RGR*	*Shoot RGR*
**Treatment**	1	1.68**	6.8**	0.013**	1.22**	35.12**	0.02**	72.62**	24.4**
**Genotype**	7	0.36**	0.68**	0.028**	0.12**	2.1**	0.004^NS^	42.95**	51.7**
Treatment*Genotype	7	0.02*	0.12**	0.01**	0.01^NS^	0.51*	0.003^NS^	9.49^NS^	12.35**
**Error**	30	0.01	0.01	0.001	0.03	0.16	0.003	7.79	3.08
Source	DF	*60 DAT Harvest*				*117 DAT Harvest*			
		*Root Zn Conc.*	*Shoot Zn Conc.*	*Root Zn Content*	*Shoot Zn Content*	*Root Zn Conc.*	*Shoot Zn Conc.*	*Root Zn Content*	*Shoot Zn Content*
**Treatment**	1	2967.3**	2468.8**	10294.6**	44469.2**	1921.2**	2112.7**	9441.35**	151925**
**Genotype**	7	95.09**	96.51**	1057.7**	2377.9**	66.46**	63.7**	523.5**	7142**
Treatment*Genotype	7	43.5**	36.85**	176.5**	10.71**	72.66**	43.13**	237.23**	3250**
**Error**	30	11.9	10.6	24.2	68.8	17.4	5.95	69.4	348
Source	DF	*Grain Yield*	*100 Seed Weight*	*No. of pods plant^-1^*	*Grain Zn Conc.*	*Grain Zn Content*	*Grain Phytate Conc.*	*[Phytate]: [Zn] Ratio*	*Estimated Zn* *Bioavailability*
**Treatment**	1	13.67**	0.32**	18135.2**	2454.02**	96510**	165.95**	6342.25**	6.08**
**Genotype**	7	3.379**	0.09**	1441.4**	75.3**	14295**	18.87**	199.9**	0.31**
Treatment*Genotype	7	1.08**	0.01^NS^	359.9^NS^	53.59**	7853**	15.54*	143.69*	0.28**
**Error**	30	0.15	0.01	214.1	7.1	282.3	4.75	45.31	0.03

DF, Degree of freedom; *, Significant (P ≤ 0.05); **, Highly significant (P ≤ 0.01); NS, Non-significant (P > 0.05); RSR, Root Shoot Ratio; RGR, Relative Growth Rate; Conc, Concentration.

The biomass partitioning (root:shoot ratio, mass basis) was significantly (P<0.01) influenced by Zn supply and among genotypes (**Table 3**). Zinc deficiency increased root shoot ratio in all genotypes except Zn-efficient genotypes (Line-11504 and Mansehra-89). Interestingly, the maximum root shoot ratio was also observed (0.57) in Zn-efficient genotype Mansehra-89, while the lower value of root shoot ratio was obtained (0.28) in Zn inefficient genotype Masoor-2006 under deficiency of Zn (**Table 3**). Zinc application in both Zn-efficient genotypes reduced RSR showing more biomass partitioning towards shoot under Zn supply.

**Table 3:** Influence of solution Zn application on root shoot ratio (at both harvest) and relative growth rate (mg g^-1^ day^-1^) in lentil genotypes.

Genotypes	Treatments	RSR		RGR	
		Harvest at 60 DAT	Harvest at 117 DAT	Root	Shoot
**Line-10502**	**Control**	0.34	0.26	9.46	14.14
	**With Zn**	0.45	0.25	2.57	12.17
**Markaz-09**	**Control**	0.40	0.28	1.85	8.45
	**With Zn**	0.41	0.26	1.07	8.68
**Masoor-2006**	**Control**	0.28	0.35	6.41	2.66
	**With Zn**	0.35	0.29	5.40	8.45
**Line-11504**	**Control**	0.50	0.34	2.03	8.61
	**With Zn**	0.47	0.25	0.57	11.39
**Masoor-85**	**Control**	0.31	0.28	6.02	8.44
	**With Zn**	0.35	0.24	4.32	10.85
**Mansehra-89**	**Control**	0.57	0.29	0.90	12.32
	**With Zn**	0.46	0.26	1.75	10.81
**Masoor-2004**	**Control**	0.33	0.25	10.57	15.11
	**With Zn**	0.42	0.27	6.46	14.31
**Shiraz-96**	**Control**	0.34	0.37	7.04	5.78
	**With Zn**	0.41	0.26	2.47	10.25
**CV (%)**		9.37	18.9	64.8	17.3
**HSD_0.05_ (T)**		0.0108 (0.005)	0.0154 (0.012)	0.8058 (0.005)	0.5067 (0.008)
**HSD_0.05_ (G)**		0.0216 (0.000)	0.0307 (0.313)	1.6117 (0.000)	1.0134 (0.000)
HSD_0.05_ (T*G)		0.0306 (0.000)	0.0435 (0.474)	2.2793 (0.324)	1.4331 (0.003)

Where Values in the parentheses represents p value; RSR, Root Shoot Ratio; RGR, Relative Growth Rate; DAT, days after transplantation.

The root relative growth rate (RGR) also showed significant variation (P<0.01) among lentil genotypes with Zn application (**Tables 2**, **3**). Application of Zn reduced the RGR in all lentil genotypes except for the Zn-efficient genotype (Mansehra-89). The maximum relative growth rate of root (10.57 mg g^-1^ day^-1^) was observed in Masoor-2004 (Zn non-responsive), which was higher with no Zn supply and the minimum value was observed (0.57 mg g^-1^ day^-1^) in the Zn-efficient genotype (Line-11504). The shoot relative growth rate had significant (P<0.01) main and interactive effects regarding lentil genotypes and treatments (**Tables 2**, **3**). The shoot relative growth rate was increased 3-fold in Zn inefficient genotype Masoor-2006 with the application of Zn. Interestingly, the higher shoot growth rate (15.1 mg g^-1^ day^-1^) was observed in the Zn non-responsive genotype (Masoor-2004) and the lower value (2.66 mg g^-1^ day^-1^) was observed in the Zn inefficient genotype (Masoor-2006) under no Zn supply (**Table 3**).

#### Grain yield

The data for the grain yield of lentil genotypes was significant (P<0.01) among the Zn treatments (**Table 1**). All lentil genotypes showed a significant increase in grain production with adequate Zn supply. As expected, the minimum grain yield (1.83 g plant^-1^) was obtained in Zn non-responsive genotype Shiraz-96 under Zn stress. The main effects showed significant variation on 100 seed weight and the number of pods per plant^-1^ (P<0.01), while the interactive effects (Treatment x genotype) showed non-significant variation (**Table 1**). The maximum 100 seed weight (2.1 g plant^-1^) and more pods per plant^-1^ (150) were calculated in Zn-efficient genotype Mansehra-89 under Zn supply, whereas the lower values were observed in Zn inefficient genotype Masoor-85 with no Zn application.

### Root and shoot Zn concentration and Zn contents

Zinc concentration in roots was improved linearly in all lentil genotypes with the application of Zn in the solution compared to the control (no Zn) at both harvests (**Table 4**). The concentration of Zn in roots varied from 23 to 54 mg kg^-1^and maximum root Zn was accumulated in the Zn-efficient genotype Mansehra-89 under Zn supply, where minimum Zn was found in Zn inefficient genotype Masoor-85 at both harvests. In 117 DAT harvest, Zinc non-responsive genotypes (Masoor-2004 and Shiraz-96) showed a minimum increase in root Zn accumulation compared to other genotypes.

**Table 4:** Effect of solution Zn application on root and shoot Zn concentrations of lentil genotypes.

Genotypes	Treatments	Root Zn Concentration		Shoot Zn Concentration	
		Harvest at 60 DAT	Harvest at 117 DAT	Harvest at 60 DAT	Harvest at 117 DAT
		mg kg^-1^	mg kg^-1^	mg kg^-1^	mg kg^-1^
**Line-10502**	**Control**	28.32 ± 2.54	31.11 ± 2.33	25.17 ± 2.43	21.36 ± 0.99
	**With Zn**	40.79 ± 0.86	43.16 ± 1.72	39.57 ± 1.99	30.8 ± 1.54
**Markaz-09**	**Control**	25.66 ± 1.2	35.26 ± 4.83	23.44 ± 1.85	23.42 ± 1.27
	**With Zn**	40.91 ± 2.63	42.44 ± 2.42	35.27 ± 2.19	33.51 ± 2.23
**Masoor-2006**	**Control**	27.36 ± 1.69	26.97 ± 2.77	27.91 ± 1.96	22.4 ± 1.37
	**With Zn**	39.1 ± 1.92	37.93 ± 2.5	39.1 ± 1.92	32.37 ± 2.23
**Line-11504**	**Control**	29.08 ± 2.93	27.88 ± 3.8	28.56 ± 3.28	21.98 ± 0.74
	**With Zn**	51.89 ± 1.34	51.08 ± 2.77	49.98 ± 1.18	43.94 ± 2.39
**Masoor-85**	**Control**	23.11 ± 1.43	29.29 ± 1.8	24.68 ± 1.24	21.58 ± 1.64
	**With Zn**	37.9 ± 2.55	40.84 ± 1.64	36.73 ± 2.13	36.85 ± 1.61
**Mansehra-89**	**Control**	29 ± 1.64	28.68 ± 3.47	28.68 ± 1.71	25.95 ± 1.03
	**With Zn**	54.06 ± 1.52	52.07 ± 2.47	51.47 ± 1.89	46.65 ± 1.76
**Masoor-2004**	**Control**	27.58 ± 2.87	30.12 ± 1.04	25.53 ± 1.49	24.9 ± 1.65
	**With Zn**	37.46 ± 2.44	37.37 ± 1.41	36.02 ± 2.35	33.82 ± 1.61
**Shiraz-96**	**Control**	25.68 ± 1.82	28.88 ± 1.99	26.68 ± 1.16	24.46 ± 1.35
	**With Zn**	39.48 ± 1.66	34.52 ± 2.79	37.25 ± 2.3	34.26 ± 1.58
**CV (%)**		9.91	11.5	9.72	8.16
***p* value (T)**		0.000	0.000	0.000	0.000
***p* value (G)**		0.000	0.004	0.000	0.000
*p* value (T*G)		0.006	0.003	0.008	0.000

Where DAT, days after transplantation.

Likewise, root Zn contents were significantly (P<0.01) increased in all lentil genotypes with Zn application (**Tables 2**, **5**). The root Zn contents varied from 13.25 µg plant^-1^ in Zn non-responsive genotype Masoor-2004 under Zn stress to 82.41 µg plant^-1^ in Zn-efficient genotype Mansehra-89 under adequate Zn supply. It was found that the Zn-responsive genotype Line-10502 and Zn-efficient genotype Line-11504 accumulated about 3-fold more Zn in root with application of Zn than in Zn inefficient or non-responsive genotypes at 60 DAT harvest. Similar results were observed in the 117 DAT harvest, though the amount of Zn contents was increased in all genotypes at adequate Zn supply.

**Table 5:** Influence of solution Zn application on root, shoot and grain Zn content of lentil genotypes under hydroponic condition.

		Zinc contents				
Genotypes	Treatments	Harvest at 60 DAT		Harvest at 117 DAT		
		Root	Shoot	Root	Shoot	Grain
		µg plant^-1^	µg plant^-1^	µg plant^-1^	µg plant^-1^	µg plant^-1^
**Line-10502**	**Control**	16.49 fg	42.88 de	32.13 d-f	84.49 de	73.6 c-e
	**With Zn**	46.59 bc	99.57 b	57.31 b-d	162.03 bc	138.7 b
**Markaz-09**	**Control**	22.13 e-g	50.55 c-e	33.77 c-f	84.32 de	75.5 c-e
	**With Zn**	52.26 b	109.12 b	58.78 bc	174.52 bc	121.3 bc
**Masoor-2006**	**Control**	16.92 fg	62.18 c-e	24.49 f	59.14 e	66.4 de
	**With Zn**	38.24 b-d	110.36 b	52.32 b-e	151.29 bc	133.3 b
**Line-11504**	**Control**	28.6 d-f	56.21 c-e	30.35 ef	73.06 de	107.4 b-e
	**With Zn**	74.66 a	152.85 a	76.36 ab	265.66 a	288.2 a
**Masoor-85**	**Control**	16.95 fg	58.03 c-e	31.11 ef	84.51 de	64.3 de
	**With Zn**	35.89 c-e	98.38 b	50.82 c-e	190.1 b	114.8 b-d
**Mansehra-89**	**Control**	38 b-d	65.35 cd	39.52 c-f	123.71 cd	88.6 b-e
	**With Zn**	82.41 a	172.48 a	87.72 a	300.59 a	315.8 a
**Masoor-2004**	**Control**	13.25 g	38.15 e	27.63 ef	93.75 de	76.7 c-e
	**With Zn**	31.34 d-f	71.59 c	46.33 c-f	158.54 bc	114.3 b-d
**Shiraz-96**	**Control**	17.72 fg	52.85 ce	29.84 ef	68.28 de	62.3 e
	**With Zn**	42.98 b-d	98.85 b	43.61 c-f	168.66 bc	105.8 b-d
**CV (%)**		13.7	18.4	9.91	13.3	13.8
**HSD_0.05_ (T)**		1.4214 (0.000)	2.3944 (0.000)	2.4048 (0.000)	5.3837 (0.000)	4.85 (0.000)
**HSD_0.05_ (G)**		2.8429 (0.000)	4.7888 (0.000)	4.8097 (0.000)	10.767 (0.000)	9.699 (0.000)
HSD_0.05_ (T*G)		4.0204 (0.000)	6.7724 (0.008)	6.8019 (0.000)	15.227 (0.000)	13.718 (0.000)

Where values in the parenthesis represents p values; DAT, days after transplantation.

The concentration of Zn in shoots was also increased significantly (P<0.01) in all genotypes under Zn supply. Lentil genotype Mansehra-89 (Zn-efficient) showed 90% more shoot Zn concentration under adequate Zn supply compared to no Zn application. The higher accumulation of Shoot Zn (51.47 mg kg^-1^) was found in Zn-efficient genotype Mansehra-89 with the application of Zn, while the lower concentration of shoot Zn 23.44 (mg kg^-1^) was observed in Zn-responsive genotype Markaz-09 under Zn stress. Similarly, shoot Zn contents significantly improved in lentil genotypes under Zn application (**Tables 2**, **5**). Shoot Zn contents ranged from 38.15-172.48 µg plant^-1^. The maximum value of shoot Zn content was found in the Zn-efficient genotype Mansehra-89 under Zn supply, while the minimum value was observed in the Zn non-responsive genotype (Masoor-2004) under Zn stress. There was 3-fold more accumulation of shoot Zn was observed in Zn-efficient genotypes (Line-11504 and Mansehra-89) with Zn application.

Shoot Zn contents had significant main and interactive effects for lentil genotypes with the application of Zn at 117 DAT harvest too (**Tables 2**, **5**). Shoot Zn contents ranged from 59 µg plant^-1^ in Zn inefficient genotype Masoor-2006 to 300 µg plant^-1^ in Zn-efficient genotype Mansehra-89. An increase of 2-3fold in root Zn contents in Zn-efficient genotype Line-11504 was calculated under Zn supply with respect to control treatment.

### Grain Zn, phytate, and Zn bioavailability

**Figure 1:**
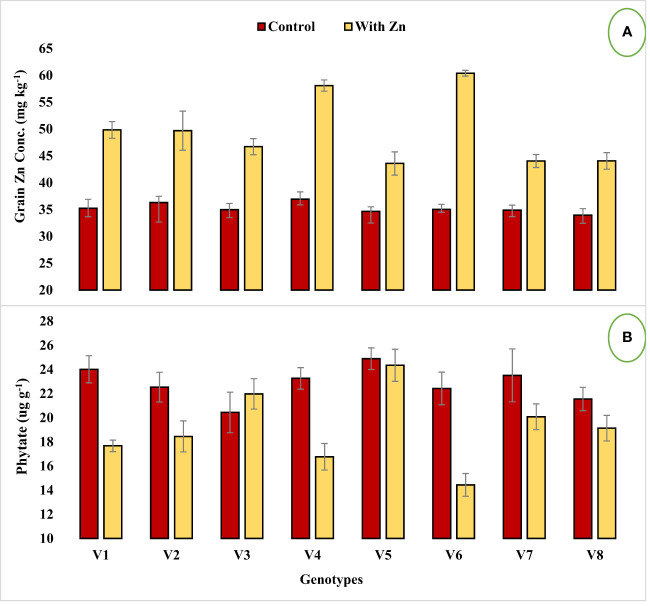
Effect of solution Zn application on **(A)** Grain Zn concentration and **(B)** Grain phytate concentration of lentil genotypes under hydroponics. Where; V1; Line-10502, V2; Markaz-09, V3; Masoor-2006, V4; Line-11504, V5; Masoor-85, V6; Mansehra-89, V7; Masoor-2004, V8; Shiraz-96. Sharing error bars are statistically at par with each other.

Genotypes differed significantly for grain Zn concentration, contents, grain phytate concentration, [phytate]:[Zn] ratio, estimated Zn bioavailability, and percent Zn bioavailability with or without Zn supply (**Table 2**). Grain Zn concentration was significantly increased in lentil genotypes with the application of Zn (**Figure 1A**). The grain Zn concentration ranged from 33.98 to 60.39 mg kg^-1^ in lentil genotypes and the maximum grain Zn was found in the Zn-efficient genotype Mansehra-89 under adequate Zn supply, while a lower value was obtained in the Zn non-responsive genotype Shiraz-96 under Zn stress. It was observed that Zn-efficient genotypes, Line-11504 and Mansehra-89 accumulated 57% and 72% more Zn in grain, respectively under Zn supply compared to control (0 Zn). The grain Zn content varied from 64 µg plant^-1^ in the Zn inefficient genotype Masoor-85 under Zn stress condition to 316 µg plant^-1^ in Zn-efficient genotype Mansehra-89 under adequate Zn supply. There was an approximate 2-fold and 3-fold increase in grain Zn accumulation in Zn-efficient genotypes Line-11504 and Mansehra-89 respectively, with Zn application.

**Figure 2:**
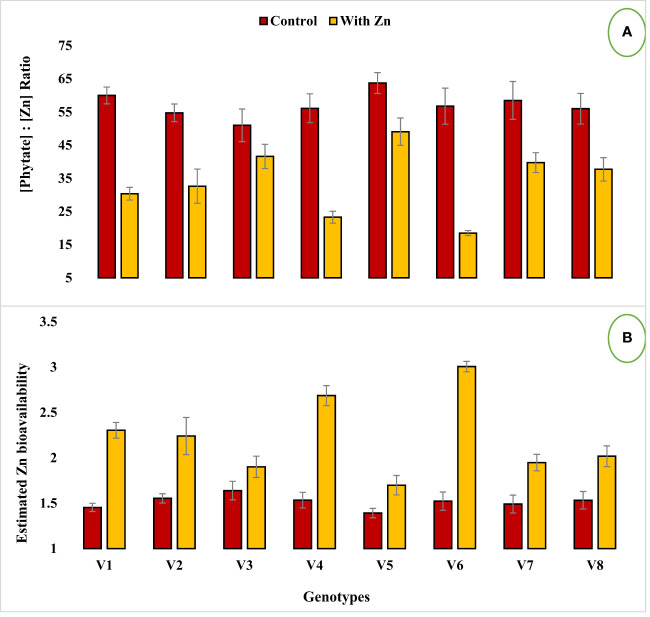
Effect of solution Zn application on **(A)** [Phytate]:[Zn] ratio and **(B)** Estimated Zn bioavailability of lentil genotypes under hydroponics. Where; V1; Line-10502, V2; Markaz-09, V3; Masoor-2006, V4; Line-11504, V5; Masoor-85, V6; Mansehra-89, V7; Masoor-2004, V8; Shiraz-96. Sharing error bars are statistically at par with each other.

**Figure 3:**
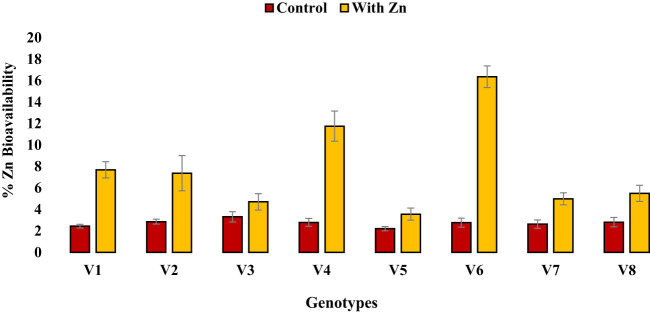
Effect of solution Zn application on percent Zn bioavailability of lentil genotypes under hydroponics. Where; V1; Line-10502, V2; Markaz-09, V3; Masoor-2006, V4; Line-11504, V5; Masoor-85, V6; Mansehra-89, V7; Masoor-2004, V8; Shiraz-96. Sharing error bars are statistically at par with each other.

Zinc application significantly (P<0.05) reduced grain phytate concentration in all genotypes (**Table 2**, **Figure 1B**) except in Zn inefficient genotypes (Masoor-2006 and Masoor-85). The grain phytate concentration varied from 14.43 µg g^-1^ (Mansehra-89 with Zn) to 24.89 µg g^-1^ (Masoor-85 at no Zn). The maximum reduction in grain phytate concentration of 28% and 35% was observed in Zn-efficient genotypes Line-11504 and Mansehra-89 under adequate Zn supply. [Phytate]:[Zn] ratio also had significant variation (P<0.01) in tested lentil genotypes with Zn application (**Table 2**, **Figure 2A**). The higher value of [phytate]:[Zn] ratio (63.71) was found in Masoor-85 (Zn inefficient) with no Zn application, while the lower value was observed (18.47) in Mansehra-89 (Zn-efficient) under Zn supply. The maximum reduction (2-fold) in [phytate]:[Zn] ratio was calculated in Zn-efficient genotype Mansehra-89, and minimum reduction was obtained in Zn in-efficient genotype Masoor-2006 under Zn supply. The bioavailability of Zn had significant variation (P<0.01) in lentil genotypes with Zn application (**Table 2**, **Figures 2B**, **3**). The maximum estimated Zn bioavailability (3.01 mg d^-1^) and percent Zn bioavailability (16.36%) were observed in Zn-efficient genotype Mansehra-89 under adequate Zn supply, while minimum values were observed in Zn inefficient genotypes.

### Relationship between various parameters of lentil genotypes

Pearson correlation coefficient (r) represents the nature of the relationship among various parameters (**Table 6**). Root dry weight had a strong positive relationship (r > 0.75 at both harvests) with shoot dry weight, with grain yield (r = 0.82), root Zn concentration (r > 0.86 at both harvests), shoot Zn concentration (r > 0.75) and grain Zn concentration (r = 0.90). It was observed that root, shoot, and grain Zn concentration had a strong positive correlation with grain yield.

**Table 6:** Pearson correlation coefficients (r) among different parameters of lentil genotypes.

	RDW1	SDW1	RDW2	SDW2	NPP	SW	GY	RZ1	SZ1	RZ2
**RDW1**										
**SDW1**	0.76									
**RDW2**	0.94	0.66								
**SDW2**	0.78	0.41	0.86							
**NPP**	0.69	0.44	0.78	0.44						
**SW**	0.84	0.33	0.80	0.70	0.73					
**GY**	0.82	0.43	0.85	0.73	0.85	0.89				
**RZ1**	0.86	0.44	0.89	0.72	0.87	0.95	0.97			
**SZ1**	0.77	0.47	0.85	0.62	0.95	0.80	0.95	0.94		
**RZ2**	0.82	0.49	0.74	0.85	0.35	0.81	0.73	0.74	0.55	
**SZ2**	0.82	0.50	0.86	0.74	0.77	0.72	0.85	0.80	0.81	0.57
**GZ**	0.90	0.54	0.86	0.75	0.73	0.95	0.93	0.96	0.85	0.88
**GP**	-0.69	-0.30	-0.67	-0.39	-0.72	-0.83	-0.60	-0.77	-0.63	-0.47
**P:Z**	-0.78	-0.39	-0.74	-0.50	-0.76	-0.93	-0.76	-0.89	-0.74	-0.65
**EZB**	0.89	0.47	0.88	0.70	0.80	0.97	0.87	0.96	0.83	0.77
**ZB**	0.92	0.52	0.95	0.80	0.81	0.94	0.90	0.97	0.87	0.79
**R:S1**	0.88	0.36	0.85	0.81	0.65	0.96	0.85	0.91	0.75	0.83
**R:S2**	0.05	0.29	0.01	-0.49	0.46	-0.04	0.01	0.08	0.24	-0.45
**RGRR**	-0.92	-0.82	-0.72	-0.56	-0.47	-0.69	-0.63	-0.67	-0.55	-0.76
**RGRS**	-0.06	-0.59	0.10	0.48	-0.09	0.27	0.19	0.17	0.05	0.30
	SZ2	GZ	GP	P:Z	EZB	ZB	R:S1	R:S2	RGRR	RGRS
**GZ**	0.72									
**GP**	-0.52	-0.71								
**P:Z**	-0.58	-0.87	0.96							
**EZB**	0.73	0.94	-0.89	-0.96						
**ZB**	0.82	0.94	-0.82	-0.90	0.98					
**R:S1**	0.79	0.90	-0.76	-0.84	0.93	0.94				
**R:S2**	0.04	-0.05	-0.34	-0.23	0.09	0.02	-0.14			
**RGRR**	-0.62	-0.78	0.55	0.67	-0.73	-0.72	-0.73	-0.12		
**RGRS**	0.15	0.12	-0.02	-0.03	0.14	0.18	0.34	-0.76	0.30	

n= 48; very strong relationship if r ≥ 0.7; strong relationship if r ≥ 0.5, week relationship if r ≤ 0.5.

RDW1, Root dry weight at 60 DAT harvest; SDW1, Shoot dry weight at 60 DAT harvest; RDW 2, Root dry weight at 117 DAT harvest; SDW2, Shoot dry weight at 117 DAT harvest; NPP, No. of pods plant^-1^; SW, 100 seed weight; GY, Grain yield; RZ1, Root Zn Conc. at 60 DAT harvest; SZ1, Shoot Zn Conc. at 60 DAT harvest; RZ2, Root Zn Conc. at 117 DAT harvest; SZ2, Shoot Zn Conc. at 117 DAT harvest; GZ, Grain Zn Conc; GP, Grain phytate Conc; P:Z, [phytate]:[Zn[Ratio; EZB, Estimated Zn bioavailability; ZB, % Zn bioavailability; R:S1, Root shoot ratio at 60 DAT harvest; R:S2, Root shoot ratio at 117 DAT harvest; RGRR, Relative growth rate of root; RGRS, Relative growth rate of shoot.

## Discussion

Biofortification is recognized as a sustainable approach to alleviating micronutrient malnutrition. Several research investigations have reported genetic variations in many crops such as wheat (Maqsood et al., 2015), rice, maize (Rasheed et al., 2019; Sanjeeva et al., 2020), cabbage, canola, and common bean (Ray et al., 2014) for Zn uptake and partitioning in grain/food. Exploitation of such genetic variation is the main basis of biofortification through breeding ventures for improving crop species even cultivars of the same species (Ortiz-Monasterio et al., 2007). These variations can be exploited to produce more efficient genotypes/cultivars promising higher yields and Zn contents in edible portions, enabling biofortification. We have reported significant variations in lentil genotypes and categorized these genotypes into four groups viz efficient, responsive, inefficient, and non-responsive (Rasheed et al., 2020). The present experiment reports differences between efficient and inefficient lentil genotypes for the growth and ionic partitioning under adequate and no Zn supply in hydroponics.

Biomass production (shoot, and root dry weights) was improved significantly in all genotypes through an increase in growth, which varied 2 to 3 fold in different categories viz efficient vs inefficient and responsive vs non-responsive genotypes (**Table 1**). The Zn efficient genotypes (Line-11504 and Mansehra-89) produced more root and shoot dry weights at both harvest and grain yield (**Table 1**). This increase was expected because Zn is an activator of a number of enzymes and its role in photosynthesis and various cell regulatory processes (Begum et al., 2016; Hidoto et al., 2017; Farouk and Al-Amri, 2019). Among lentil genotypes, grain yield was 2-fold higher in Mansehra-89 under adequate Zn supply in comparison to control treatment (**Tables 1**, **2**). A significant improvement in the grain yield of lentils and other crops with the application of Zn has also been documented by various researchers (Hussain et al., 2011; Zou et al., 2012; Maqsood et al., 2015; Ram et al., 2015; Rasheed et al., 2020).

In this study, the lentil genotypes varied significantly for Zn contents in their root, shoot, and grains (**Table 5**). Lentil genotype Mansehra-89 (Zn-efficient) had maximum root (54 mg kg^-1^) and shoot (51.5 mg kg^-1^) Zn concentration under adequate Zn supply. The differences in Zn concentration and content in tissues of plants may be attributed to variations in their Zn uptake capacity (Hussain et al., 2011; Rasheed et al., 2020), root-to-shoot transport of absorbed Zn (Gupta et al., 2016; Rehman et al., 2018) and Zn sequestration (Cakmak and Kutman, 2018; Rasheed et al., 2019) (**Tables 3**-**5**). The differences in the accumulation of Zn depend upon the function of intracellular binding sites and uptake capacity (Vymazal and Brezinova, 2015). Differences in Zn concentration in different tissues may be attributed to the growth dilution effect as biomass production possibly due to higher enzymatic activity (Pal et al., 2019) Root activity is also an important factor having high availability and capacity of Zn uptake by converting the exchangeable Zn form to available form through the release of organic acids and ion exchange, which ultimately contribute to higher crop yield and quality (Gupta et al., 2016). Zinc accumulation in grains is not only related to root uptake but also depends on the internal remobilization and redistribution within the different plant parts (Jiang et al., 2007), which mostly occurs through transport in the xylem, transfer from the xylem to the phloem, and re-translocation in the phloem. This is evident from the positive correlation of the relative growth rate of root with grain Zn concentration and [phytate]:[Zn] ratio (**Table 6**).

In the present experiment, the Zn concentration in root and shoot varied significantly at the vegetative stage (60 DAT) and maturity (117 DAT) (**Table 4**). The higher root and shoot concentration at the early stage compared to crop maturity indicated the re-translocation of absorbed Zn, and can be attributed to increased biomass at maturity (**Table 4**). Several researchers documented the significant variations in Zn accumulation at the vegetative stage, flowering stage, and after the flowering stages (Waters and Grusak, 2008; Samardjieva et al., 2014). The regulation of Zn concentration in different plant parts may be influenced by shoot biomass, leaf area, number of leaves, and distribution of Zn across metal sinks. According to Gupta et al. (2016), a tissue with a higher concentration of Zn may have lower Zn accumulation due to its low biomass. Moreover, Zn translocation from shoots and roots toward grains is the possibility of lower Zn concentration in these organs at the maturity stage compared to the vegetative growth stage. The higher Zn contents in grains, means higher the Zn translocation from shoot and roots resulting in higher biofortification.

The grain Zn contents varied significantly among lentil genotypes under the application of Zn (**Table 5**, **Figure 1**). There was a 3-fold increase in grain Zn contents in Zn-efficient genotypes (Line-11504 and Mansehra-89) under adequate Zn supply compared with no Zn application. Previous studies also documented that Zn application had a positive effect on grain Zn contents (Hidoto et al., 2017; Rasheed et al., 2020). The grain Zn contents were improved in lentil genotypes depending on their potential under adequate Zn supply. The higher grain Zn contents were calculated in Mansehra-89 (316µg plant^-1^) compared to other genotypes under Zn application, and this characteristic of Mansehra-89 makes it more Zn efficient and responsive.

Many substances such as the antinutrients (phytate) present in the edible portion reduce the Zn bioavailability to humans (Brown et al., 2001). Phytate is the most important inhibitor of the absorption of Zn, which is present in most plant foods, particularly cereals, pulses, and legumes, as it influences the absorption efficiency of micronutrients from diet (Hambidge et al., 2010). Its presence in dietary food forms insoluble complexes with Zn, which reduces digestion or absorption due to the unavailability of intestinal phytate enzymes in humans (Jiang et al., 2007). Increased grain Zn contents by Zn application improved its bioavailability (**Table 5**) which is evident from the reduced [phytate]:[Zn] ratio in grains. We found a strong negative correlation between grain Zn concentration with grain phytate concentration (r = -0.71) and [phytate]:[Zn] ratio (r = -0.87) (**Table 6**). It is therefore considered a significant criterion for screening the crop genotypes for biofortification (Hussain et al., 2011; Hussain et al., 2013; Maqsood et al., 2015; Cakmak and Kutman, 2018; Rasheed et al., 2020).

In the present study, Zn application significantly reduced the grain phytate concentration in all genotypes except Zn inefficient genotypes (**Figure 1**). The lower values of grain phytate concentration were observed in Zn efficient genotypes (Line-11504 and Mansehra-89). The maximum reduction (35%) in phytate was calculated in Mansehra-89 (Zn efficient genotype) under adequate Zn supply. This may be the effect of the growth dilution effect in Zn efficient genotypes, which produced more biomass under Zn application, hence the phytate contents were reduced (Thavarajah et al., 2009; Johnson and Thavarajah, 2013). Moreover, a similar kind of reduction in grain phytate concentration was previously reported by Erdal et al. (2002) and Rasheed et al. (2020). This reduction in the concentration of phytate might be due to changes induced by Zn in P uptake from the root zone and the subsequent translocation of P within the plant body (Huang et al., 2002). On the other hand, Zn inefficient genotypes (Masoor 2006 and Masoor 85) had higher phytate contents relative to their lower biomass under Zn supply. A strong negative correlation (r = -71) between grain phytate concentration and grain Zn concentration (**Table 6**) also supports the antagonistic effect as reported by Hussain et al. (2013). However, the Zn efficient genotypes (Line-11504 and Mansehra-89) have more ability to utilize Zn in favor of reducing grain phytate concentration as compared to Zn inefficient genotypes.

The presence of grain phytate adversely affects minerals (especially Zn) and causes a reduction in its absorption into the human body (Hussain et al., 2011; Rasheed et al., 2020). However, only its concentration is not significant and its ratio with Zn ([Phytate]:[Zn]) is considered a good indicator for estimating the bioavailability of Zn. For better absorption within the human intestine, [Phytate]:[Zn] ratio should be lower than 20 (Weaver and Kannan, 2002; Hussain et al., 2013). The ratio among [Phytate]:[Zn] varied significantly at both Zn levels and the maximum ratio was observed in Masoor-85 (64) with no Zn application, while the minimum ratio was observed in Mansehra-89 (18.5) under Zn supply (**Figure 2**). Zinc application significantly reduced the [phytate]:[Zn] ratio under Zn supply. Lentil genotype Mansehra-89 reduced the grain [phytate]:[Zn] ratio to the desired level of 18 with an application of Zn (**Figure 2**). We also found a strong negative correlation between grain phytate concentration (r = -0.89) and [phytate]:[Zn] ratio (r = -0.96) with estimated Zn bioavailability (**Table 6**). The physiological requirement of Zn for an adult person is almost 3 mg/day, which is required to be absorbed (net) into the human body (Institute of Medicine, 2001; Hotz and Brown, 2004). Daily consumption of food should fulfill the physiological requirement of Zn, for regular nutrition of an adult with Zn. According to some recent research, homeostasis of Zn in the human intestine documented the application of the trivariate model for the measurements of Zn bioavailability in the human daily diet (Hambidge et al., 2010). The trivariate model evaluates the bioavailability of Zn from the daily food intake by using the total intake amount of phytate and Zn in the daily diet. Distinct from [phytate]:[Zn] ratio, the use of the mathematical Zn absorption model explains the quantitative measurements of Zn bioavailability by utilizing the absorption of Zn into the body (Miller et al., 2007). As the human body cannot store enough Zn (Rick, 2000), there should be adequate estimated Zn bioavailability (>3 mg) in the daily intake amount of lentils (100 g) for the requirement of daily Zn for improving the human diet. Mansehra-89 (Zn-efficient genotype) had the required amount of estimated Zn bioavailability (3.01 mgd^-1^) compared to other genotypes under the Zn application (**Figures 2**, **3**).

## Conclusions

The application of Zn fertilizer is necessary to increase crop yield and its quality as most soils of the world are deficient in Zn. However, the uptake of Zn by roots and its translocation to different parts of the plant, especially in grains, is dependent on soil Zn availability and the morphological characteristics of various crops. Significant genetic variation exists among the various lentil genotypes (Zn-efficient, inefficient, Zn-responsive, and non-responsive) under Zn supply. Among different parts of the lentil, the root has accumulated relatively more Zn with respect to shoot and grains under Zn supply. There was a 3-fold increase in grain Zn contents in Zn-efficient genotypes (Line-11504; 288.2 µg plant^-1^ and Mansehra-89; 316 µg plant^-1^) under adequate Zn supply. Lentil genotype Mansehra-89 also reduced the grain [phytate]:[Zn] ratio to the desired level with the application of Zn and had the required amount of estimated Zn bioavailability (3.01 mgd^-1^) compared to other genotypes. This information may be utilized and incorporated in future breeding ventures to develop new genotypes of lentils and other crops for improving the nutrition of people who are undernourished.

## Data availability statement

The original contributions presented in the study are included in the article/supplementary material. Further inquiries can be directed to the corresponding author.

## Author contributions

NR: Investigation, Methodology, Writing – original draft. MAM: Conceptualization, Data curation, Funding acquisition, Supervision, Writing – review & editing. TA: Conceptualization, Data curation, Methodology, Supervision, Writing – review & editing. MA: Formal analysis, Methodology. IS: Formal analysis, Investigation, Methodology. SE: Data curation, Formal analysis, Investigation. AN: Writing – review & editing. HB: Formal analysis, Writing – review & editing. MX: Writing – review & editing.
